# Fast *in vitro* protocol for the visualization and quantitative high-throughput analysis of sprouting angiogenesis by confocal microscopy

**DOI:** 10.1016/j.xpro.2021.100690

**Published:** 2021-09-13

**Authors:** Lanette Kempers, Ivo van der Bijl, Anne-Marieke D. van Stalborch, Bas Ponsioen, Coert Margadant

**Affiliations:** 1 Sanquin Research and Landsteiner laboratory, Amsterdam University Medical Center, 1066 CX Amsterdam, The Netherlands; 2 Molecular Cancer Research, Center for Molecular Medicine, University Medical Center Utrecht, Utrecht University, 3584 CX Utrecht, The Netherlands; 3 Angiogenesis Laboratory, Department of Medical Oncology, Cancer Center Amsterdam, Amsterdam University Medical Center, 1081 HV Amsterdam, The Netherlands

**Keywords:** Cell Biology, Cell culture, Cell-based Assays, Developmental biology, High Throughput Screening, Microscopy, Molecular Biology

## Abstract

We describe an optimized, cost-effective, reproducible, and robust protocol to study sprouting angiogenesis in glass-bottom 96-well plates by confocal microscopy, ideal for screening of drug or shRNA libraries. Effective and stable knockdown of gene expression in primary endothelial cells is achieved by lentiviral transduction. Dynamic behavior of individual cells and fluorescent proteins is analyzed by time-lapse imaging, while competitive advantages in tip cell formation are assessed using mixtures of differentially labeled cell populations. Finally, we present a macro for high-throughput analysis.

For complete information on the use and execution of this protocol, please refer to van der Bijl et al. (2020) and Kempers et al. (2021).

## Highlights

• High-throughput microscopy analysis of sprouting angiogenesis for drug/shRNA screening
• Analysis of dynamic cell and protein behavior during sprouting by time-lapse microscopy
• Mosaic assays to image competitive advantages in tip cell behavior
• Macro for fast automated quantitative analysis

## Graphical abstract

**Figure:**
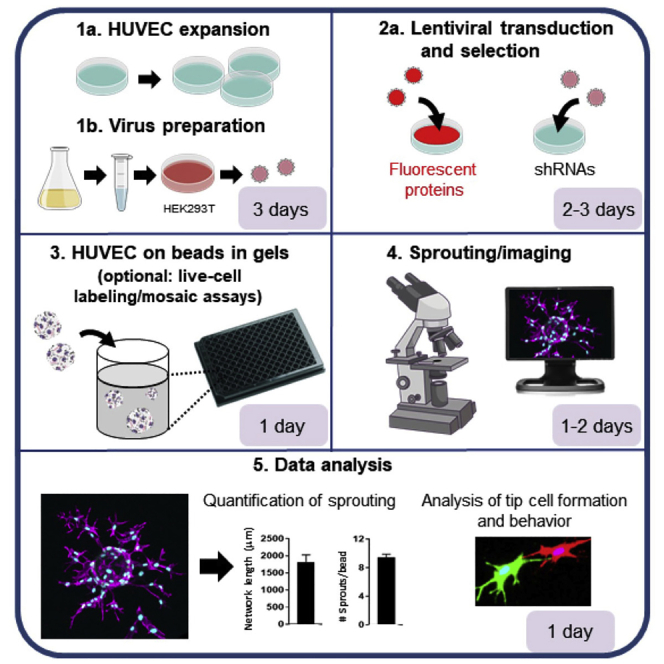

## Before you begin

### Experimental design considerations

**Figure 1:**
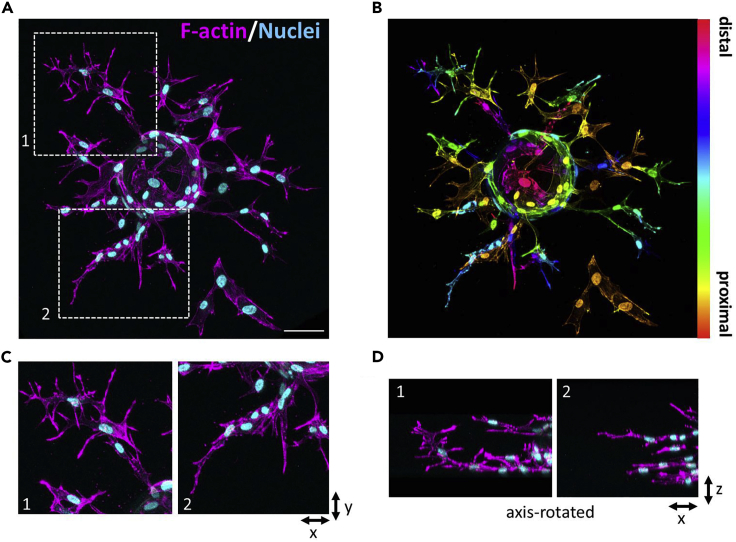
Assessment of sprouting angiogenesis by confocal microscopy (A) Maximum projection of confocal z-stack comprised of 142 z-slices showing sprouting after 48 h, stained with phalloidin to visualize actin filaments (F-actin; pseudocolored *magenta*) and Hoechst to stain the nuclei (pseudocolored *cyan*). Bar, 75 μm. (B) Depth coding (using LUT *Spectrum*) of the same stack of images. (C and D) Zooms of insets 1 and 2, showing tip cell morphology in more detail.

The current protocol is designed to achieve fast and robust human umbilical vein endothelial cell (HUVEC) sprouting in fibrin gels, and to visualize sprouting and tip cell characteristics, such as the formation of filopodia, by confocal microscopy (Figure 1). With the described cell culture set-up, maximal numbers of HUVECs are obtained with a limited number of culture steps, thus allowing to perform sprouting screens within the life-span of primary endothelial cells. Instead of freshly isolating endothelial cells we use commercially available pools of HUVECs, which we expand to the extent that the same batch of cells can be used for a large number of conditions during consecutive experiments. The described sprouting assay allows co-culture with several other cell types and can be used as a platform for the screening of clinical samples as well as drug or short hairpin (sh) RNA libraries, thus providing a unique and versatile set-up for both fundamental research into the regulation of angiogenesis and screening of patient material for diagnostic and therapeutic goals. Homogeneous and stable suppression of target gene expression using shRNAs is achieved by lentiviral transduction followed by puromycin selection, which quickly generates a population of cells that can be used for functional assays during a week. Building on previously established protocols to analyze sprouting angiogenesis (Eglinger et al., 2017; Nakatsu and Hughes, 2008; Nowak-Sliwinska et al., 2018), the assay described here is performed in ‘half-area’ 96-well glass-bottom plates, which require small volumes of reagents, allow fast, robust, and highly efficient sprouting already within 20–48 h, and are suitable for medium- to high-throughput screening. While the focus here is primarily on the initial phases of sprouting, we perform this assay in the absence of fibroblasts, which are required for lumenization (Newman et al., 2011). Furthermore, we also describe how to perform live-cell labeling in this set-up, using cell-permeable fluorescent dyes or expression of fluorescent proteins, as well as mosaic sprouting assays with a mixture of differentially labeled cells. Important criteria for selecting the right kind of microscope, especially when performing live imaging, include phototoxicity, speed/temporal resolution and sensitivity. While we have used confocal systems for the applications described here, confocals are relatively slow and can damage the sample, compared to some other systems. This can be overcome with resonant scanning, at the cost of reduced resolution. Alternatives to confocal systems include light sheet microscopes and two-photon microscopes. While the former have little phototoxicity and are ideal for the imaging of fast dynamics, the latter have superior confocality and penetration depth but are potentially harmful because of the high energy input. As different research questions will require specific adjustments to experimental design, we also present some alternatives and modifications to the protocol and conditions described here.

### Prepare cell culture materials and buffers

**Timing: ~4 h**

1. Prepare mammalian cell culture media as described in the tables below.

**Table:** Preparation of Dulbecco’s Modified Eagle’s Medium (DMEM)

Reagent	Final concentration	Amount
DMEM	n/a	440 mL
FBS	10%	50 mL
200 mM L-Glutamine	2 mM	5 mL
D-Glucose	4.5 g/L	2.25 g
100 mM Sodium pyruvate	1 mM	5 mL
Penicillin	100 U/mL	
Streptomycin	100 μg	
**Total**	**n/a**	**500 mL**

DMEM complete medium can be stored for up to 6 months at 2°C–8°C.

**Table:** Preparation of Endothelial Growth Medium-2 (EGM-2)

Reagent	Final concentration	Amount
EBM-2	n/a	440 mL
Bullet kit	10%	50 mL
200 mM L-Glutamine	2 mM	5 mL
Penicillin	100 U/mL	
Streptomycin	100 μg	
**Total**	**n/a**	**500 mL**

EGM-2 complete medium can be stored for up to 4 months at 2°C–8°C.

**Table:** Preparation of Iscove’s Modified Dulbecco’s Medium (IMDM)

Reagent	Final concentration	Amount
IMDM	n/a	440 mL
FBS	10%	50 mL
200 mM L-Glutamine	2 mM	5 mL
Penicillin	100 U/mL	
Streptomycin	100 μg	
**Total**	**n/a**	**500 mL**

IMDM complete medium can be stored for up to 3 months at 2°C–8°C.

2. Coat cell culture flasks and/or dishes with fibronectin (1 μg/mL) or gelatin (0.1%).

***Note:*** Coating with fibronectin is efficient at 37°C for 60 min, gelatin requires only 10 min. HEK293T cells are poorly adherent and fibronectin coating will facilitate their adhesion and proliferation. HUVEC culture is stimulated by gelatin coating.

***Note:*** For coating of tissue culture plastic for HEK293T cells, fibronectin can also be substituted by serum, which contains large quantities of fibronectin and vitronectin that will be absorbed by the plastic and stimulate integrin-mediated cell adhesion. Serum can be re-used multiple times for this purpose.

3. Prepare buffers and beads as described in the tables below.

**Table:** Preparation of PBS with MgCl2/CaCl2

Reagent	Final concentration	Amount
PBS	n/a	1 L
MgCl2	0.5 mM	
CaCl2	1 Mm	
**Total**	**n/a**	**1 L**

PBS with MgCl2/CaCl2 can be stored for up to 12 months at 15°C–30°C.

**Table:** Preparation of 4% paraformaldehyde (PFA)

Reagent	Final concentration	Amount
PBS	n/a	1 L
PFA	4%	40 g
**Total**	**n/a**	**1 L**

PFA can be stored for up to 12 months at −20°C.

***Note:*** PFA is flammable, corrosive, toxic, carcinogenic and is an irritant for the skin, eyes, and the respiratory tract. A lab coat, gloves, face mask, and safety goggles should be worn when handling PFA. All steps should be performed inside a fume hood, and waste should be disposed of in a special container.

**CRITICAL:** PFA should be dissolved first in 800 mL PBS inside a fume hood, while continuously stirring. Add NaOH until the solution clears, filter it, adjust the pH to 7.0 with HCl and fill up to 1 L with PBS. Aliquot the solution for long-term storage at −20°C.

**Table:** PBS/gelatin preparation

Reagent	Final concentration	Amount
PBS	n/a	1 L
Gelatin	0.1%	1 g
**Total**	**n/a**	**1 L**

PBS-gelatin can be stored for up to 6 months at 4°C.

***Note:*** PBS-gelatin should be sterilized before use in tissue culture, for instance using bottle top filters from Millipore (pore size 0.22 μm).

**Table:** Bead suspension

Reagent	Final concentration	Amount
Cytodex beads	20 g/L	1 g
PBS	n/a	50 mL
**Total**		**50 mL**

The beads can be stored for up to 12 months at 20°C–22°C.

***Note:*** This will generate a stock solution of approximately 3 × 10^6^ beads/L. The beads should be sterilized before use in an autoclave at 120°C.

### HUVEC expansion and storage

**Timing: 1 week**

4. Warm cell culture media to 37°C before use.
5. Add a large volume of IMDM containing 10% fetal bovine serum, 2 mM L-glutamine, and 1% penicillin/streptomycin (~400 μL/cm^2^) to a T25 culture flask.

***Note:*** We use IMDM here only for economical reasons. Since this medium is discarded (see step 11 below) we prefer to not use EGM-2 for this step.

6. Place the flask at 37°C and 5% CO_2_.
7. Thaw 1 Lonza cryovial of HUVECs, carefully aspirate cell suspension, and gently pipet into a coated T25.
8. Distribute the cells over the culture flask by gentle rocking.
9. Place the flask at 37°C and 5% CO_2_ for ~2 h.
10. 2 h after seeding, check whether the cells have adhered and have started to spread over the surface.
11. Replace the IMDM with warm EGM-2 (~200 μL/cm^2^).
12. After 2 days, remove the EGM-2 medium and wash cells with a large volume of 37°C PBS.
13. Add Trypsin/EDTA (~10 μL/cm^2^) and distribute over entire surface.
14. Place cells back at 37°C and 5% CO_2_ for 1 min.
15. Check under microscope whether cells have rounded up and/or detached.
16. Gently tap flask to fully detach rounded cells.
17. Add Trypsin Neutralization Solution (10–20 μL/cm^2^) for ~30 s.
18. Add EGM-2 and resuspend cells.
19. Seed cells into a coated T150.
20. After 2 days, trypsinize the cells as described above and transfer to coated T150s (usually 4–6), depending on cell density.
21. After 2 days, freeze cells for cryo storage, 25 cm^2^ of confluent cells per vial (this yields usually 30–40 vials).

**CRITICAL:** Make sure HUVECs grow sufficiently (troubleshooting 1**)**

### Preparation of constructs for lentiviral transduction

**Timing: 2–3 days**

Constructs containing shRNAs are obtained from the TRC MISSION library. Each MISSION shRNA clone is constructed within the lentivirus plasmid vector pLKO.1-puro, containing ampicillin and puromycin resistance genes for selection in bacterial or mammalian cells, respectively, and transformed into *Escherichia coli* (DH5α). Individual clones are purchased as frozen bacterial glycerol stocks containing Terrific Broth (TB), carbenicillin (100 μg/mL), and 15% glycerol, which can be stored at −80°C in 96-well plates. For isolation of plasmid DNA, bacteria are inoculated in Lennox Broth (LB) containing Ampicillin (100 μg/mL) and grown for 12–16 h at 37°C. The plasmid DNA is purified using the NucleoBond Xtra Midi- or Maxiprep protocol (Macherey-Nagel), according to the manufacturer’s instructions. Constructs needed for lentiviral production are purified from *Eschirichia coli* Stbl3, grown at 30°C. Required constructs include:

*pHDM-HgpM2 GAG/POL*, encoding the main structural viral proteins and reverse transcriptase

*pRC-CMV-REV1B*, encoding the post-transcriptional regulator Rev1b necessary for efficient synthesis of viral proteins,

*pHDMG-G VSV ENV*, coding for the envelope protein of Vesicular Stomatitis Virus,

*pHDM-TAT 1B*, encoding the Tat1b protein, which facilitates viral entry into target cells

*pLKO.1-puro* encoding the shRNA of interest and puromycin resistance for selection of positive target cells

***Note:*** Sequence confirmation of the insert after plasmid isolation is advised. The following reagents and conditions are optimized for sequencing of shRNA-encoding inserts:

**Table:** Reaction mix for sequencing reaction

	Stock	Final	Dilution	1 RXN
H2O	1×	0.5×	2	6
DMSO	1×	0.05×	20	1
Betaine	5M	1M	5	4
Big Dye Terminator Buffer	5×	1×	5	4
Big Dye terminator	5×	1×	5	4
Primer	10 μM	0.25 μM	40	0.5
DNA	1 μg/mL	0.025 μg/mL	40	0.5

**Table:** PCR cycling conditions

Steps	Temperature	Time	Cycles
Initial denaturation	98	1 min	1
Denaturation	98	30 s	
Annealing	54	10 s	40
Extension, 3 s increment	60	2–4 min	

### Production of lentivirus-like particles encoding shRNAs

**Timing: 5 days**

22. Seed human embryonic kidney (HEK) 293T cells 1:4 in fibronectin-coated filter-cap flasks.
23. The following morning, transfect HEK293T cells with constructs as in Table 1. The cells should be 50%–80% confluent.

**Table 1:** Transfection mix for the production of lentivirus-like particles

*Trans*IT + TAT		cm^2^	cm^2^	
**Surface area per condition**	**Ratio**	1	**75**	
*pHDMG·G VSV ENV*	2.0	44	4950	Ng
*pHDM·HgpM2 GAG/POL*	1.0	22	1667	Ng
*pRC-CMV-REV1B*	1.0	22	1667	Ng
*pHDM-TAT1B*	1.0	22	1667	Ng
*pLKO.1-puro* encoding shRNA of interest	13.0	289	21667	Ng
**Total #ng of DNA/cm**^**2**^	18.0	**400**	30000	Ng
**Total #μL of *Trans*IT/cm**^**2**^		**0.40**	30	mL
Optimem		10.00	750	mL
24. Dilute TransIT-LT1 (0.4 μL/cm^2^) in Optimem (10 μL/cm^2^) and mix thoroughly.
25. Add viral constructs (44 ng/cm^2^ *pHDMG-G VSV ENV*, 22ng/cm^2^ *pHDM-HgpM2 GAG/POL*, 22 ng/cm^2^ *pRC-CMV-REV1B*, 22 ng/cm^2^ *pHDM-TAT1B*) to obtain a mastermix.
26. Add mastermix (10.4 μL/cm^2^) to a tube containing *pLKO.1-puro* coding for desired shRNA (289 ng/cm^2^).
27. Leave transfection mix at 20°C–22°C for approximately 20 min in the dark.

**CRITICAL:** Do not incubate the transfection mixture longer than 30 min.

28. Add transfection mix to the HEK293T cells, place in the incubator at 37°C in the presence of 5% CO_2_.
29. Remove medium containing the transfection mix ~6 h later, and replace with approximately 100 μL/cm^2^ of DMEM.
30. Place cells back in the incubator at 37°C and 5% CO_2_.
31. At 48 h after transfection, harvest the culture medium, centrifuge at 500 × *g* for 5 min, store at 4°C.
32. At 72 h after transfection, harvest the culture medium, centrifuge at 500 × *g* for 5 min.
33. Mix with the 48 h harvest, filter using a 0.45 μm pore filter, aliquot and store at −80°C.

**CRITICAL:** Protocol steps 28–33 must be performed in a BSL-2 lab. Tight adherence to BSL-2 regulations on sample handling and the disposal of plastic ware and media is required.

**CRITICAL:** A good viral titer is crucial for efficient transduction (troubleshooting 2)

## Key resources table

**Table:** 

REAGENT or RESOURCE	SOURCE	IDENTIFIER
**Bacterial and virus strains**		

*Eschirichia coli* DH5α	Thermo Fisher Scientific	N/A
*Eschirichia coli* Stbl3	Thermo Fisher Scientific	N/A

**Chemicals, peptides, and recombinant proteins**		

Aprotinin	Sigma-Aldrich	Cat#A1153
Betaine	Sigma-Aldrich	B0300
CaCl2	Merck	CAS 10035-04-08
CellTracker Green	Thermo Fisher Scientific	Cat#C2925
CellTracker Red	Thermo Fisher Scientific	Cat#C34552
D-Glucose	Sigma-Aldrich	CAS 50-99-7
Dimethyl sulfoxide (DMSO)	J.T.Baker	CAS 67-68-5
Dulbecco’s Modified Eagle’s Medium (DMEM)	Thermo Fisher Scientific	Cat#41965-039
EDTA Triplex III	Merck	CAS 6381-92-6
Endothelial Basal Cell Growth Medium-2 (EBM-2)	PromoCell	C-22211
Endothelial Cell Growth Medium-2 with supplements (EGM-2)	PromoCell	C-22011
Fetal Bovine Serum (FBS), heat inactivated	Bodinco	5010
Fibrinogen (Haemocomplettan P)	CSL Behring	Cat#B02BB01
Fibronectin	Sigma-Aldrich	Cat#F1141
Gelatin	Sigma-Aldrich	Cat#G1890
L-Glutamine	Sigma-Aldrich	Cat#G7513
Hoechst 33342	Thermo Fisher Scientific	Cat#H3570
Iscove’s Modified Eagle Medium (IMDM)	Thermo Fisher Scientific	Cat#12440053
MgCl2	Sigma-Aldrich	CAS 7791-18-6
Opti-MEM Reduced Serum Medium, GlutaMAX Supplement	Thermo Fisher Scientific	Cat#51985034
Phosphate-buffered saline (PBS), pH 7.0	Fresenius Kabi	N/A
Paraformaldehyde (PFA)	Merck	Cat#1.04005
Phalloidin, Texas Red-conjugated	Thermo Fisher Scientific	Cat#T7471
Penicillin/Streptomycin	Sigma-Aldrich	P0781
Polyethylene glycol (PEG) 6000	Sigma-Aldrich	CAS 25322-68-3
Puromycin	InvivoGen ant-pr-1	ant-pr-1
Sodium pyruvate	Thermo Fisher Scientific	Cat# 11360070
Thrombin	Sigma-Aldrich	Cat#T1063
*Trans*IT-LT1	Mirus Bio	MIR2360
Triton X-100	Sigma-Aldrich	Cat#T8787
Trypsin Neutralization Solution	Lonza	Cat# CC-5002
Trypsin	Sigma-Aldrich	Cat#59418C
VEGF-A (VEGF165), human recombinant	R&D Systems	Cat#293-VE

**Experimental models: cells**		

Human embryonic kidney (HEK) 293T cells	ATCC	CRL-3216
Human umbilical vein endothelial cells (HUVECs)	Lonza	C2519A

**Oligonucleotides**		

*pLKO.1-puro* encoding the shRNA of interest and puromycin resistance for selection of positive target cells	TRC Mission Library	N/A
Control sequence/scrambled sequence	TRC Mission Library	Cat# C002
*Primer: U6 Forward:* 5′-GGACTATCATATGCTTACCG-3′	IDT	Custom Oligo

**Recombinant DNA**		

*pHDM-HgpM2 GAG/POL*, encoding the main structural viral proteins and reverse transcriptase	Addgene	164441
*pRC-CMV-REV1B*, encoding the post-transcriptional regulator Rev1b necessary for efficient synthesis of viral proteins	Addgene	164443
*pHDMG-G VSV ENV*, coding for the envelope protein of Vesicular Stomatitis Virus	Addgene	164440
*pHDM-TAT 1B*, encoding the Tat1b protein, which facilitates viral entry into target cells	Addgene	164442

**Software and algorithms**		

Imaging software pertaining to confocal microscope (used here: Leica LAS X)	https://www.leica-microsystems.com/products/microscope-software/p/leica-las-x-ls/	N/A
Optional: *Navigator* module in Leica LAS X		N/A
ImageJ/Fiji	https://imagej.net/Downloads	Schindelin et al., 2009
*Sprout Morphology* plugin	https://imagej.net/Sprout_Morphology	Eglinger et al., 2017
Macro “*Automated sprout analysis*”, generated here	https://github.com/Sprouting-angiogenesis/Automated-sprout-analysis	N/A
Optional: *3D viewer* plugin	https://imagej.nih.gov/ij/plugins/3d-viewer/	N/A
Microsoft Excel	https://www.microsoft.com/nl-nl/microsoft-365/excel	N/A
Optional: GraphPad Prism 5.0	http://www.graphpad.com/scientific-software/prism	N/A

**Other**		

Cytodex 3 microcarrier beads	Sigma	Cat#C3275
96-well Half Area High Content Imaging Glass Bottom Microplate	Corning	Cat#4580
0.45 μm Pore filters	Whatman (GE Healthcare)	Cat#10462100
0.2 mL Low Profile Thin-walled 8 Tube & Flat Cap Strips	Thermo Scientific	AB-0773
BigDye Terminator v3.1 Cycle Sequencing Kit	Thermo Fisher Scientific	4337456
Bottle top filters	Merck Millipore	S2GVU05RE
Thermocycler	MJ Research	Dyad Disciple
Autoclave	N/A	N/A
DNA sequencing system	Thermo Fisher Scientific	3730 DNA Analyzer
Multichannel pipette	N/A	N/A
Cell counter	Innovatis	CASY TT
Confocal setup with climate chamber, multi-position mode, and water pump	Leica SP8	N/A

## Materials and equipment

In addition to the abovementioned reagents and materials (key resources table), a BSL-1 cell culture facility with incubator and flow cabinet, and including standard tissue culture disposables, is required. Furthermore, a bacterial lab equipped with shaking incubator, as well as materials and reagents for transformation and plasmid isolation is needed. A cell counter (for instance; CASY TT) is used to assess the numbers of endothelial cells to be incubated with Cytodex beads, while a confocal microscope equipped with a CO_2_-supplemented climate chamber, as well as a time-lapse and multi-position module, is required for live imaging. We use a Leica SP8 confocal set-up, but any other confocal microscope with these specifications will be effective. For the production of lentiviral particles and the lentiviral transduction of endothelial cells with shRNAs, BSL-2 facilities are required. Finally, for the analysis, quantification, and storage of data, FIJI software with the indicated plugins, as well as our newly generated macro “*Automated sprout analysis*” (key resources table) are required, as well as a powerful computer with a fast processor, plenty of hard-disk space, and sufficient RAM (at least 16 GB).

***Alternatives:*** Instead of Hoechst, DAPI can be used. The CellTracker dyes used here for live imaging can also be substituted for others. A number of different cell-permeable dyes, coupled to a variety of fluorophores, exist that are retained in the cytosol or label distinct subcellular compartments. While this protocol is optimized for the 96-well ‘half area’ plates, the use of imaging plates of different size and/or well number is also possible. The volumes indicated here should then be adjusted accordingly. Finally, other endothelial cells can be used instead of HUVECs, although not all endothelial cells grow and sprout equally efficiently.

## Step-by-step method details

### Endothelial cell culture and lentiviral transduction for stable knockdown

**Timing: ~1 week**

1. Thaw 1 vial of HUVECs and seed into a gelatin-coated T75.
2. After 1 or 2 days, HUVECs can be replated as described above into appropriate number of coated T150s, usually 3–4, depending on the pool of HUVECs (some pools grow better than others). This step is for experiments that require large numbers of cells. Otherwise, skip this step and proceed with 3.
3. After 1 or 2 days, seed HUVECs for lentiviral transduction into coated 6-well plates at 40%–60% confluency, 1 or 2 wells per condition.
4. The following day, transduce HUVECs with virus-like particles, obtained as described above.

***Note:*** HUVECs can be transduced with individual shRNAs or with pools of different shRNAs targeting the same gene. The latter is suitable for initial screening purposes. The efficiency of knockdown varies per shRNA, as do the chances of off-target effects. Furthermore, it is recommended to use 2 or 3 different clones per target gene, to reduce the chances of erroneous interpretation of results due to off-target effects. Finally, a scrambled sequence in the same vector should be used as a control.

5. HUVECs are incubated for 12–16 h with the shRNA lentivirus solution (1 mL/well of a 6-wells plate) in DMEM, supplemented with 0.2–1.0 mL of EGM-2.

***Note:*** Supplement with EGM-2, depending on virus concentration and/or efficiency. It is advised to use as little virus as possible to achieve efficient knockdown of target gene expression. Since the shRNA-coding sequence is randomly integrated into the host genome, increasing copy number increases the chance of destroying critical genes, which will affect the cell in ways other than by knockdown of the target gene. Titration of the virus (with puromycin) is recommended.

**Figure 2:**
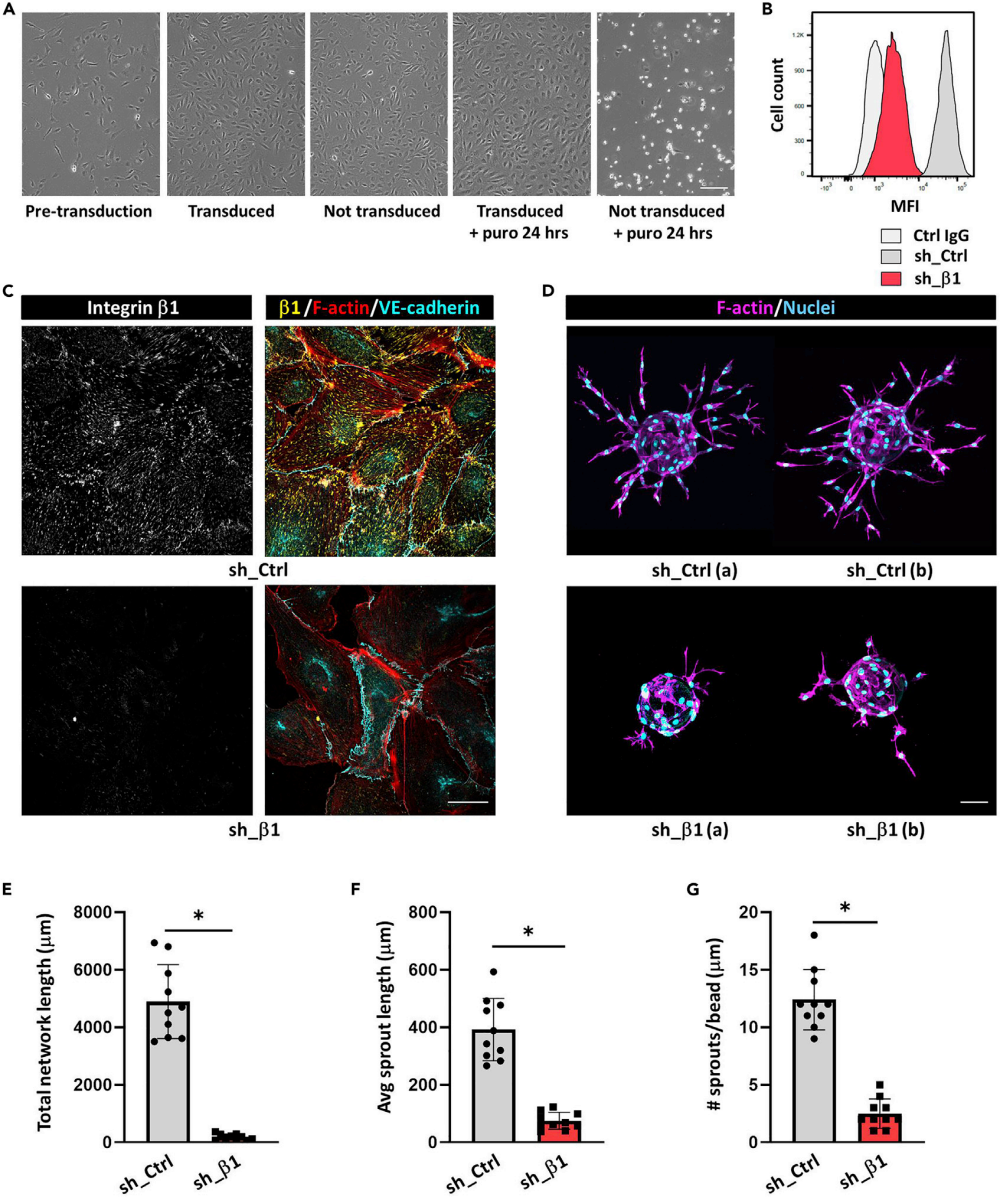
Generation of HUVECs with stable suppression of gene expression and assessment of the efficiency of knockdown (A) Phase/contrast images showing HUVECs before and after lentiviral transduction with pLKO.1 and selection with puromycin. Untransduced controls are all dead after two days of puromycin treatment. Bar, 100 μm. (B) Determination of knockdown efficiency using flow cytometry. Histograms represent cell-surface levels of β1 integrin in HUVECs expressing scrambled sequences (sh_Ctrl) or shRNAs against integrin β1 (sh_β1). Bar, 20 μm. (C) Confocal images of HUVECs stained for β1 integrin (*yellow*), F-actin (*red*), and VE-cadherin (*gray*). (D–G) (D) Composite images showing examples of sprouting efficiency in fibrin gels. Bar, 75 μm. Total network length/bead (E), average sprout length (F), and number of sprouts/bead (G) were determined using the protocol described here from 10 beads out of 2 experiments. Data are means ± SEM, statistically significant differences are denoted by ∗ p < 0.05, (unpaired two-tailed t test).
7. After 2 days, the selected cells can be used for attachment to beads (see sprouting assay protocol below).
8. The efficiency of knockdown should be assessed in parallel by flow cytometry (for surface proteins) or Western blotting (for intracellular proteins), using validated antibodies (see Figure 2). If these are not available, knockdown can be confirmed by Q-PCR.

***Note:*** Selection should be finished 2–3 days after the addition of puromycin. Always take 1 well of non-transduced cells along to confirm puromycin-induced cell death. In case the selection is suboptimal, a kill curve should be performed first to establish the ideal puromycin concentration.

6. Replace the medium with EGM-2 containing puromycin (1 μg/mL) to select positive cells (see Figure 2).

**CRITICAL:** Make sure sufficient cells after obtained after selection (troubleshooting 3).

### Assay to screen for factors regulating sprouting angiogenesis

**Timing: 1–2 days**

This step describes how to achieve fast and robust HUVEC sprouting in fibrin gels, and to visualize sprouting and tip cell characteristics, such as the formation of filopodia, by confocal microscopy. Work in the flow hood to keep cells and materials sterile.

9. Prepare beads
  a. Resuspend beads.
  b. Transfer 250 μL of resuspended beads to a sterile 1.5 mL Eppendorf tube.
  c. Allow beads to settle and remove supernatant.
  d. Add 250 μL of EGM-2.
10. Prepare cells
  a. Harvest HUVECs by trypsinization and resuspend in EGM-2.
  b. Count cells using a cell counter.
  c. Adjust cells to a concentration of 660.000 cells/mL (=1 × 10^6^ cells/1.5mL).

***Note:*** This is sufficient to coat approximately 1200 beads. To coat less or more beads, change numbers accordingly.

11. Attach cells to beads
  a. Add 1.5 mL of cell suspension to a 50 mL tube (=1 × 10^6^ cells in total).
  b. Resuspend beads and add 40 μL to the cell suspension.
  c. Place cell/bead mixture at 37°C and 5% CO_2_ for 4 h.
  d. Resuspend every 20 min.

12. Incubation of beads with cells
  a. Add cell/bead mixture to T25.
    ***Note:*** Don’t fill up the pipette with the entire 1.5 mL; too many cells/beads will stick to the inside of the pipette.
  b. Add 3.5 mL of EGM-2 to T25.
  c. Place cell/bead mixture at 37°C and 5% CO_2_ for 12–16 h.

13. Harvest HUVEC-coated beads
  a. Resuspend beads and transfer to a 50 mL tube.
  b. Rinse T25 once with 3 mL of EGM-2 and add to the same tube.
  c. Allow beads to settle.

***Note:*** The knockdown of some target genes may affect HUVEC adhesion to Cytodex beads. Visually inspect whether HUVEC adhesion is efficient.

14. Wash HUVEC-coated beads
  a. After beads have settled, remove the supernatant, add 1 mL of EGM-2, resuspend and allow beads to settle. Repeat.

15. Prepare fibrin gel
  a. Add 0.6 mL of 10 mg/mL fibrinogen to 2.4 mL of PBS, then add 18 μL of 15 U/mL aprotinin.
  b. After removing the supernatant from the beads, add fibrinogen/aprotinin/PBS mixture to the 50 mL tube.
  c. Pipet a drop of 1,875 μL of a 10 U/mL thrombin solution in the middle of a well of a glass-bottom 96 well plate.
    ***Note:*** The plates mentioned here require small volumes of reagents, ideal for testing multiple conditions simultaneously, and the glass bottom is ideal for confocal imaging. However, plates with different properties (such as different well size, or plastic bottom if phase/contrast imaging is used instead of confocal microscopy) can also be used.
  d. Add 30 μL of the fibrinogen/aprotinin/PBS mixture containing HUVEC-coated beads and resuspend once, very gently.

***Note:*** Avoid air bubbles. Do not move plate.

**Figure 3:**
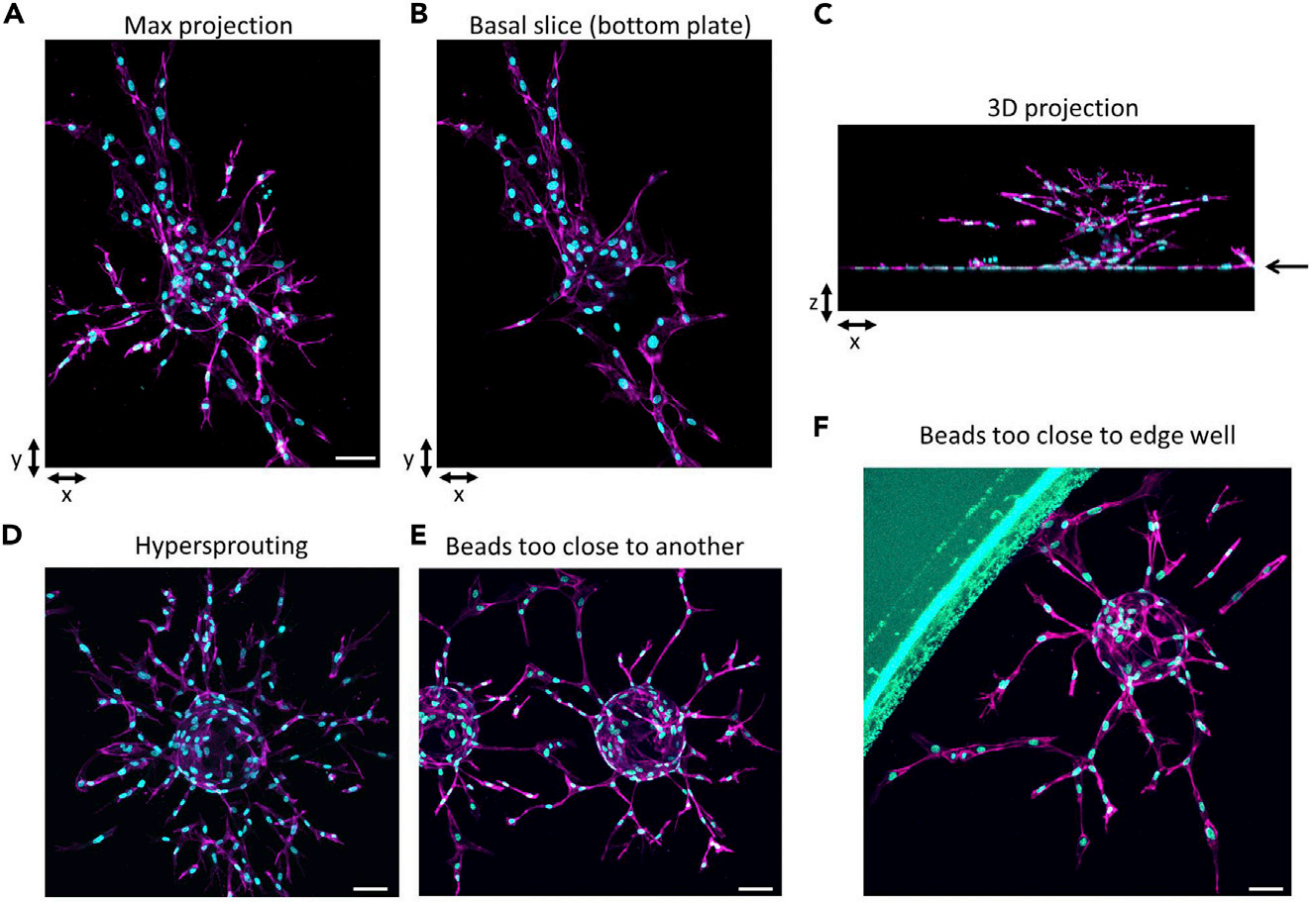
Common problems associated with the assay Maximum projection of an image stack (A), basal z-slice only (B), and 3-D projection along the x-axis (C), showing HUVECs spreading on the bottom of the plate (*arrow*). Example of hypersprouting (D), beads too close to each other (E) and beads too close to the well periphery (F). All stainings show F-actin (*magenta*) and Hoechst (*cyan*). Maximum projections are generated from stacks of 199-291 z-slices. Bars, 75 μm.

**CRITICAL:** Make sure the beads are properly resuspended and distributed. While too many beads per well or beads that are too close to one another could be good to study anastomosis, it hinders a proper discrimination of sprouts belonging to individual beads (see Figure 3).

16. Polymerization
  a. Allow the fibrin gel to polymerize for 5 min in the flow hood at 20°C–22°C.
  b. Gently place plate in incubator for 15 min.

***Note:*** Avoid air bubbles. Do not move plate!

17. Sprouting
  a. In the flow hood, very gently drip 100 μL of EGM-2 on top of the gel.
  b. Place HUVEC/bead/gel mixture at 37°C and 5% CO_2_ for 20–48 h.

**CRITICAL:** The relative quantities of thrombin and fibrinogen are crucial for the polymerization and quality of the gels. While poor gel quality can affect sprouting in various ways, a common problem is that beads sink to bottom and cells start to adhere to the bottom of the well (see Figure 3) (troubleshooting 4).

***Note:*** The optimal window required for sprouting should be determined first for appropriate experimental conditions. We occasionally observe excessive sprouting already within 48 h, which complicates the analysis and quantification (see Figure 3). Also, it will be more difficult to identify stimulatory effects on sprouting under these circumstances (troubleshooting 5).

### Multi-position confocal imaging of fixed material

**Timing: 8–12 h**

After the desired time of sprouting (in glass-bottom ‘half-well’ 96-well plates, efficient sprouting is already visible within 20–48 h after initiation of the assay), cells can be fixed for immunofluorescence analysis. For the visualization of individual sprouts and cells, rather than subcellular structures, we use phalloidin to stain actin filaments, and Hoechst to stain nuclei. Both stains are very bright and because of their small size, their delivery to cells in the fibrin gels is much more efficient than that of antibodies. The time required for imaging of an entire plate will depend on the numbers of beads to be imaged per condition, as well as microscope settings such as number of z-slices, resolution, and scan speed.

18. Staining
  a. Aspirate the medium and wash once with PBS containing MgCl2/CaCl2.
  b. Fix with 500 μL of 4% PFA for 15 min at 20°C–22°C.
  c. Wash 4 times with PBS.
  d. Permeabilize with 500 μL of 0.5% Triton X-100 in PBS for 5 min at 4°C (this is sufficient for robust delivery of phalloidin, in contrast to antibodies which would require longer permeabilization).
  e. Block non-specific binding with 5% BSA in PBS containing MgCl2/CaCl2 for 2 h at 20°C–22°C.
  f. Wash the gels 3 times with PBS containing MgCl2/CaCl2.
  g. Incubate with Hoechst and phalloidin for 12–16 h at 4°C.
  h. Wash gels 3 times with PBS containing MgCl2/CaCl2 for 10 min and store at 4°C until imaging. Although it is advised to image the plates shortly after staining, the plates can be stored for a number of weeks.
    ***Note:*** In case of storage, keeping the phalloidin in until imaging increases the quality of staining.

***Optional:*** For the visualization of subcellular structures or specific proteins, stainings with antibodies can be performed. For details on immunostaining in fibrin gels see: (Eglinger et al., 2017; Nakatsu and Hughes, 2008)

**Figure 4:**
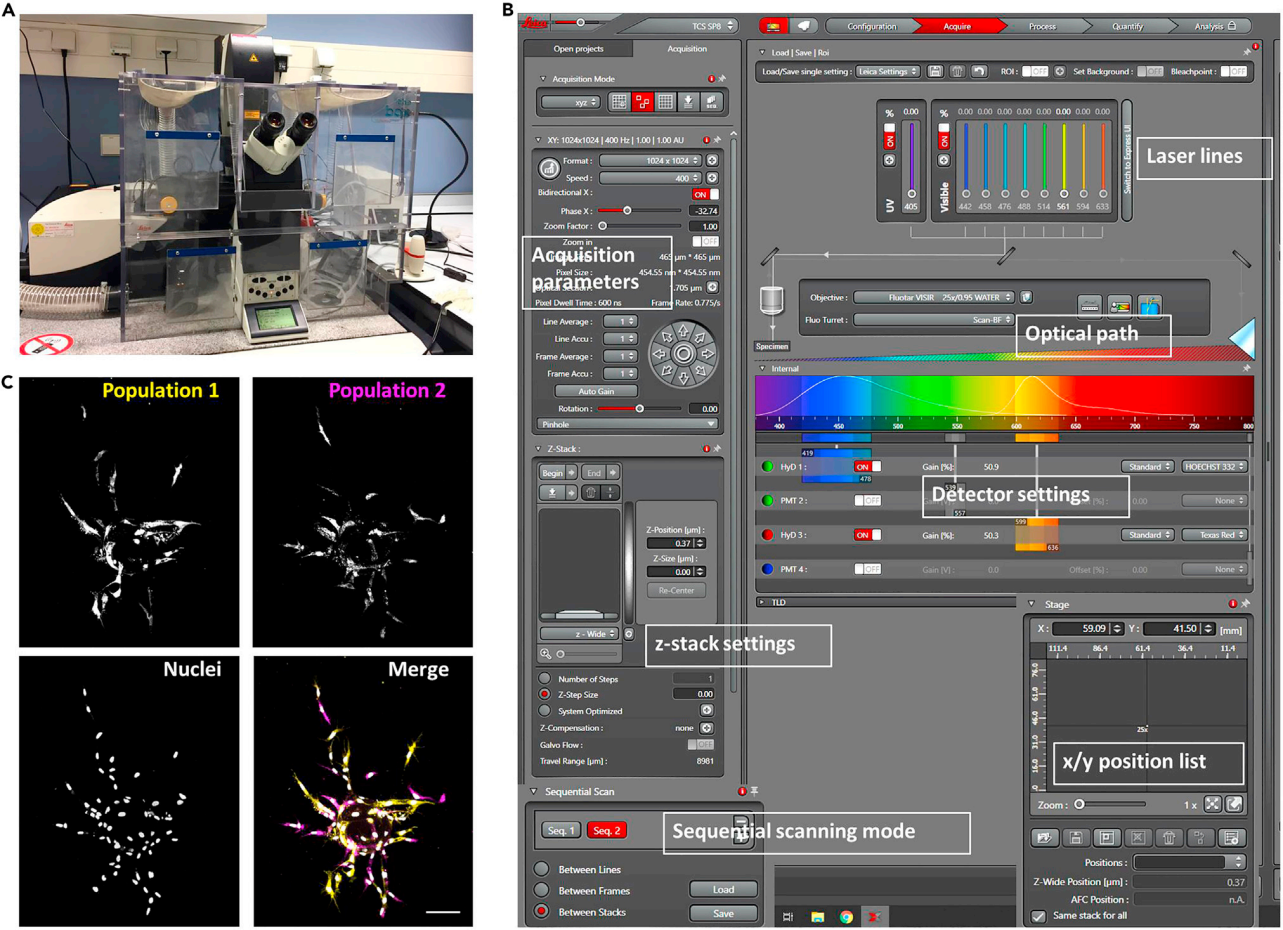
Confocal imaging and mosaic sprouting assays (A) The confocal set-up used for assays described in this paper requires a module for multi-position imaging and a 37°C climate chamber equipped with CO_2_ and humidifier, as well as a water pump for prolonged water immersion imaging. (B) Screenshot of confocal settings for imaging of sprouts in multi-position mode. (C) Example of a mosaic assay for simultaneous assessment of differential sprouting properties between conditions. Two different HUVEC populations are differentially labeled using CellTracker dyes (pseudocolored *yellow* and *magenta*), mixed in a 1:1 ratio on beads, and allowed to sprout in fibrin gels, whereafter they are fixed and the nuclei stained (*gray*). Images are maximum projections of confocal z-stacks comprised of 183 slices. Bar, 75 μm.
  e. Optimize laser and gain settings.
  f. Set the appropriate number of z-slices. Example: at a 1.5 μm z-slice thickness, a typical bead with sprouts of about 300 nm in height will require around 200 z-slices.
  g. Run a pilot stack to confirm or adjust settings.
  h. Select xyz positions and save to positions list. Attempt to image ~10 beads per condition.
    ***Note:*** The settings mentioned here will enable fast imaging with high-quality results, but several settings including the number of z-slices should be adjusted according to individual demands on image quality or imaging speed.
  i. Start the experiment. The positions list can be created at the end of the day to let the confocal run for 12–16 h.

***Note:*** The above settings will generate high-quality images with great resolution, but imaging with these settings will take on average 15 min per bead. For the analysis of large numbers of beads, scanning time can be reduced to about 3 min per bead using resonant scanning. The latter option will result in lower resolution but will still generate sufficient detail for proper visualization and quantification.

***Note:*** Because of bead-to-bead heterogeneity and variability in sprouting, it is advised to image multiple beads (5–10) per condition. Potential bias in selecting the beads for imaging could be reduced by performing the selection in a blind manner. Furthermore, unbiased bead selection may be aided using microscope applications that scan the entire well at low magnification (such as Leica Navigator), thus giving an impression of the general efficiency of sprouting per condition. Finally, phase/contrast images can be acquired of the same plate in parallel to confirm differences in sprouting between conditions.

19. Multi-position confocal imaging
  a. Apply two drops of water to the lens of a 20× long-distance water objective, as well as a drop of water on the bottom of the 96-well plate underneath the wells to be filmed.
  b. Place plate on the stage of confocal microscope.
    ***Note:*** The use of objectives with long working distance is preferred. Best results are obtained with water objectives, which generate less optical aberrations at long working distance, as compared to oil-immersion objectives. Moreover, oil immersion objectives are less suitable for multi-position imaging, as the oil will be distributed all over the plate, increasing the risk of focus loss. For longer imaging periods with water objectives, the use of a water pump is required to maintain water immersion. Use no higher magnification than 20–25× if entire beads with all pertaining sprouts are to be imaged.
  c. Select the appropriate laser lines (for instance, excitation at 405 nm for Hoechst, and 561/594 nm for Texas Red-conjugated phalloidin).
  d. Determine laser/gain and other confocal settings, for instance image dimensions 1024 × 1024 pixels, 0.75 μm/pixel, pinhole 1 μm, scan speed 400 Hz, bidirectional scanning, and performing sequential scanning between stacks (see Figure 4).

### Cell labeling and mosaic sprouting assays to assess differences in tip cell formation

**Timing: 1–2 days**

While the protocol described above allows for visualization of tip cell morphology in detail (see Figure 1), differences in tip cell behavior or competitive advantages in tip cell formation can be assessed more directly in mosaic assays with differentially labeled cell populations, e.g., by using CellTracker as described below.

20. Labeling of HUVECs with cell-permeable CellTracker dyes
  a. Trypsinize HUVECs of experimental condition and control.
  b. Count HUVECs and resuspend equal numbers in EGM-2.
  c. Add a different CellTracker dye to each population, for instance CellTracker Green for the experimental condition, and CellTracker Red for the control (both at suggested concentrations of 0.1 μM).
  d. Wash the cells twice with large volumes of PBS.

***Note:*** Perform these steps in the flow hood to keep cells and dyes sterile. CellTracker dyes are dissolved in DMSO as a 1000× solution, according to the manufacturer’s instructions.

**Figure 5:**
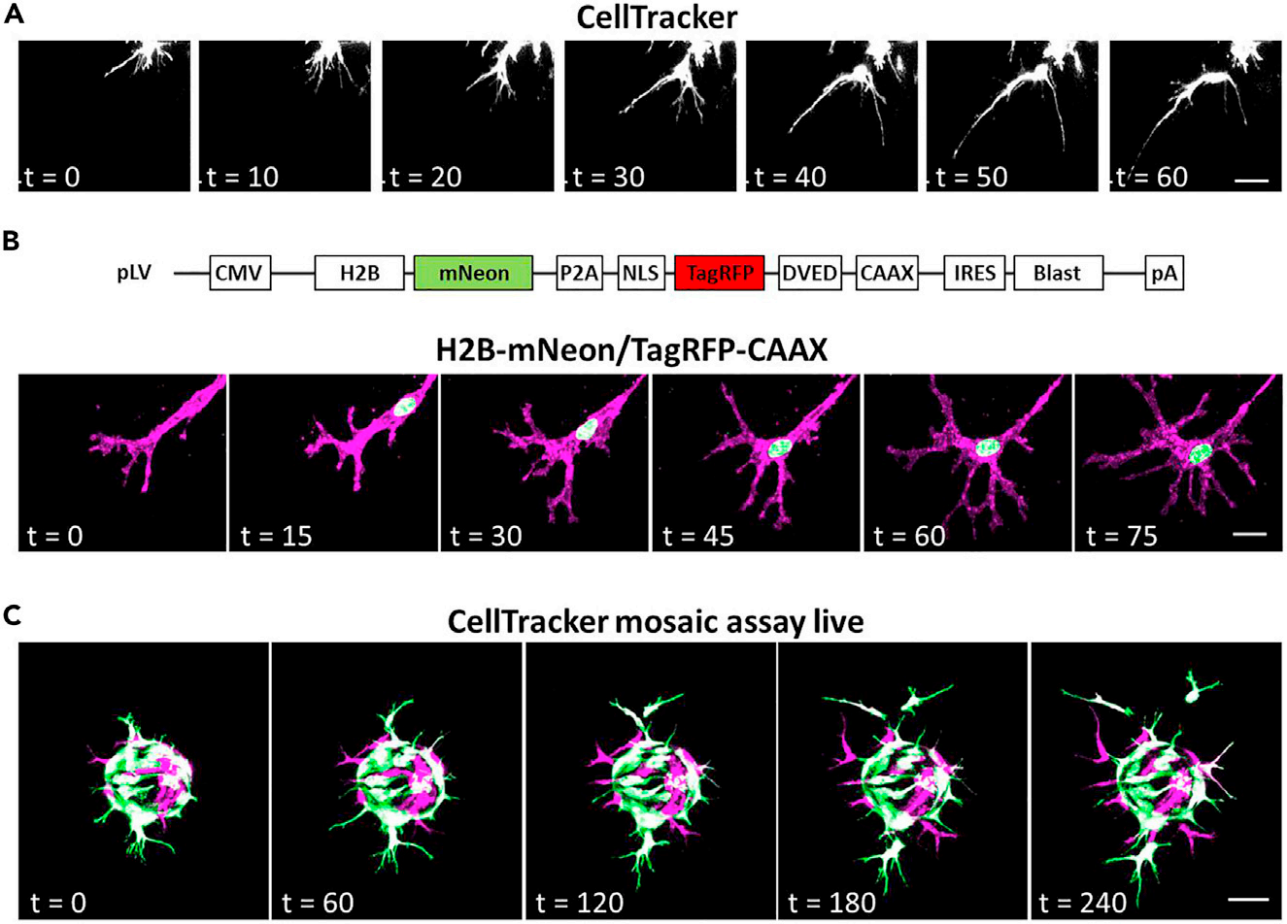
Live imaging approaches for visualization of tip cell behavior during sprouting (A) Stills from a single-channel time-lapse movie showing filopodial dynamics in sprouting HUVECs labeled with CellTracker. Images were acquired every 10 min with 61 z-slices per time-point. Bar, 20 μm. (B) Schematic representation of the construct used for live imaging (*top*). Stills from a pseudocolored dual-channel time-lapse movie, showing a tip cell expressing H2B-mNeon to visualize the nucleus, and TagRFP-CAAX to track the plasma membrane and endomembranes (*bottom*). Images were acquired every 15 min with 66 z-slices per time-point. Bar, 10 μm. (C) Stills from a pseudocolored dual-channel time-lapse sequence showing tip cell dynamics in a mosaic sprouting assay with a 1:1 mixed population of HUVECs differentially stained with distinct CellTracker dyes. Imaging started 20 h after sprouting, images were acquired from 99 z-slices with a 20 min interval. Bar, 75 μm.

***Note:*** CellTracker dyes are cell-permeable but once in the cell, they are converted into cell-impermeable reaction products. They are transferred to daughter cells after division. CellTracker Green produces uniform cell labeling, while CellTracker Red prominently labels vesicular compartments (see Figures 4 and 5).

***Note:*** Wear protective clothing, gloves, and eye/face protection when handling DMSO or the DMSO dye solution. DMSO easily penetrates the skin and facilitates the absorption of organic molecules into tissues. Dispose of waste and materials in compliance with the appropriate local regulations.

21. Attaching labeled HUVECs to beads
  a. Pre-incubate for 30 min in the dark at 37°C.
  b. Mix the two populations in a 1:1 ratio.
  c. Incubate labeled HUVEC mixture with the beads for 12–16 h at 37°C as described in steps 11 and 12.
  d. Harvest beads with labeled HUVECs as described in steps 13 and 14.
  e. Add beads with labeled HUVECs to fibrin gels and start sprouting assay as described in steps 15–17.

22. Fix cells at appropriate time-point, e.g., at 20 h after the induction of sprouting.

**CRITICAL:** CellTracker dyes can induce toxic effects in the long run. Determine cellular health by visual inspection and decide on the optimal time-point to fix the cells. For tip cell competition assays, formation of large sprouts is not necessary and the experiment can be fixed at an early time-point.

23. Label cells after fixation with Hoechst as described above in step 18. Keep plates in the dark to avoid loss of CellTracker signal.
24. Select the appropriate laser lines on a confocal set-up (for instance; 405 nm for Hoechst, 488 nm for CellTracker Green, and 591 nm for CellTracker Red).
25. Image the cells as described above (step 19).
26. Create maximum projections of the z-stacks as described in the quantification and statistical analysis section below. The separate channels will reveal cells of each population, as well as the nuclei of all cells (see Figure 4C).
27. Quantification of tip cells is performed by counting all tip cells for each color, and dividing this number by the total number of cells in that color. This will correct for potential differences in total cell numbers in each condition.

**CRITICAL:** First determine the best conditions for labeling (ie concentration, incubation times) with CellTracker dyes (troubleshooting 6). Note that prolonged incubation increases dye dilution due to cell division, as well as transfer of dyes to adjacent cells.

**CRITICAL:** To rule out (indirect) effects of the dye on tip cell formation it is recommended to include a condition where the different CellTracker dyes are swapped between cell populations. Furthermore, it is advised to include a condition in which control cells, labeled with either dye, are mixed in a 1:1 ratio. The number of tip cells in each color should be around 50% in this condition.

### Live imaging by time-lapse confocal microscopy

**Timing: 1–2 days**

In addition to imaging fixed materials, this protocol describes how to generate time-lapse movies from sprouting cells in fibrin gels, which will reveal the dynamic behavior of tip cell filopodia and of individual cells within sprouts. To visualize endothelial cells, they can be labeled using CellTracker dyes, or engineered to express a fluorescent protein. As an example of the latter, we here use a bicistronic construct in a lentiviral vector, encoding mNeonGreen labeled histone 2B (H2B) as well as a CAAX motif tethered to tagRFP (Bolhaqueiro et al., 2018). The C-terminal CAAX motif used here is from K-Ras and integrates specifically in the plasma membrane, thus marking the cell membrane and endocytic/pinocytic compartments derived thereof, while H2B marks the chromatin. Therefore, this approach is useful for the simultaneous imaging of membranes and nuclei in living cells (see Figure 5).

28. Label cells with CellTracker dyes as described above in step 20.

Or:

29. Follow steps 22–32 of the section “Production of lentivirus-like particles”, but using the lentiviral expression vector of interest, instead of shRNA-encoding *pLKO.1-puro.*
30. Transduce HUVECs as in steps 1–5 of section “Endothelial cell culture and lentiviral transduction“.
31. If desired, select positive cells with antibiotics as described in step 6 of section “Endothelialcellculture and lentiviral transduction“.

***Note:*** We use a lentiviral vector here for the delivery of our fluorescent protein of interest. Any marker protein can be used in this manner, within the size limit of a lentiviral vector (~10 kilobases in total; virus production is less efficient for inserts > 2kb).

***Note:*** If imaging with lentivirally delivered proteins is initiated shortly after transduction, a BSL-2 imaging facility is required.

***Note:*** CellTracker is easily combined with stable suppression of gene expression using shRNAs. To achieve robust expression of a fluorescent marker protein in stable knockdown cells, we recommend transducing and selecting shRNA-expressing cells first, followed by transduction to express the marker protein. A different antibiotic (for example blasticidin) could be used to select marker protein-expressing cells, but this will often not be necessary given the high degree of transduction efficiency using lentiviral systems, and the fact that positive cells can easily be selected by visual inspection.

32. Attach the fluorescent HUVECs to beads as described in step 21.
33. Determine the best time-point to start imaging. Generally, sprouting does not occur earlier than 16–18 h after placing the beads in the gels.
34. Replace the medium approximately 1 h before starting to set up the microscope. The microscope should be prewarmed and atmospherically stabilized at 37°C and 5% CO2.
35. Select the appropriate laser lines and settings. Appropriate time intervals will depend on the hypothesis, e.g., imaging filopodia dynamics will require shorter intervals than investigating tip cell sprouting or sprout elongation.
36. Start the experiment as described above in step 19.

***Note:*** Because CellTracker can fade, impose toxicity, or transfer between cells during prolonged incubation, it is advisable to not image CellTracker-loaded cells too long.

**CRITICAL:** It is important to first determine the optimal confocal settings (number of z-slices per position, intervals between images), as well as potential phototoxicity and bleaching effects (troubleshooting 7).

## Expected outcomes

The protocol described here will achieve robust sprouting of endothelial cells in a short time-frame, followed by detailed visualization and quantitative assessment of sprouting characteristics such as length and numbers of sprouts, numbers of individual cells, etc. The ‘half-area’ 96-well plates require limited amounts of reagents and are suitable for medium- to high-throughput screening. Dynamic behavior of individual cells within sprouts can be monitored using fluorescent probes by time-lapse microscopy, while the mosaic assays will assess relative sprouting efficiencies and competitive advantages between control and experimental conditions in the same well.

## Quantification and statistical analysis

**Timing: 5 min–5 h**

The protocol below makes use of our newly generated macro “*Automated sprout analysis*” that reiterates sprouting analysis using the FIJI/ImageJ plugin *Sprout morphology*, based on a previously described method (Eglinger et al., 2017). Upon importing raw confocal data, the macro will create a maximum projection, despeckle the image, and enhance the contrast by 0.3%. Subsequently the macro will detect beads, sprouts and nuclei according to adjustable settings and store the results in a separate folder. The required time will depend on the number of beads analyzed, binning options, and the computer used.

**Figure 6:**
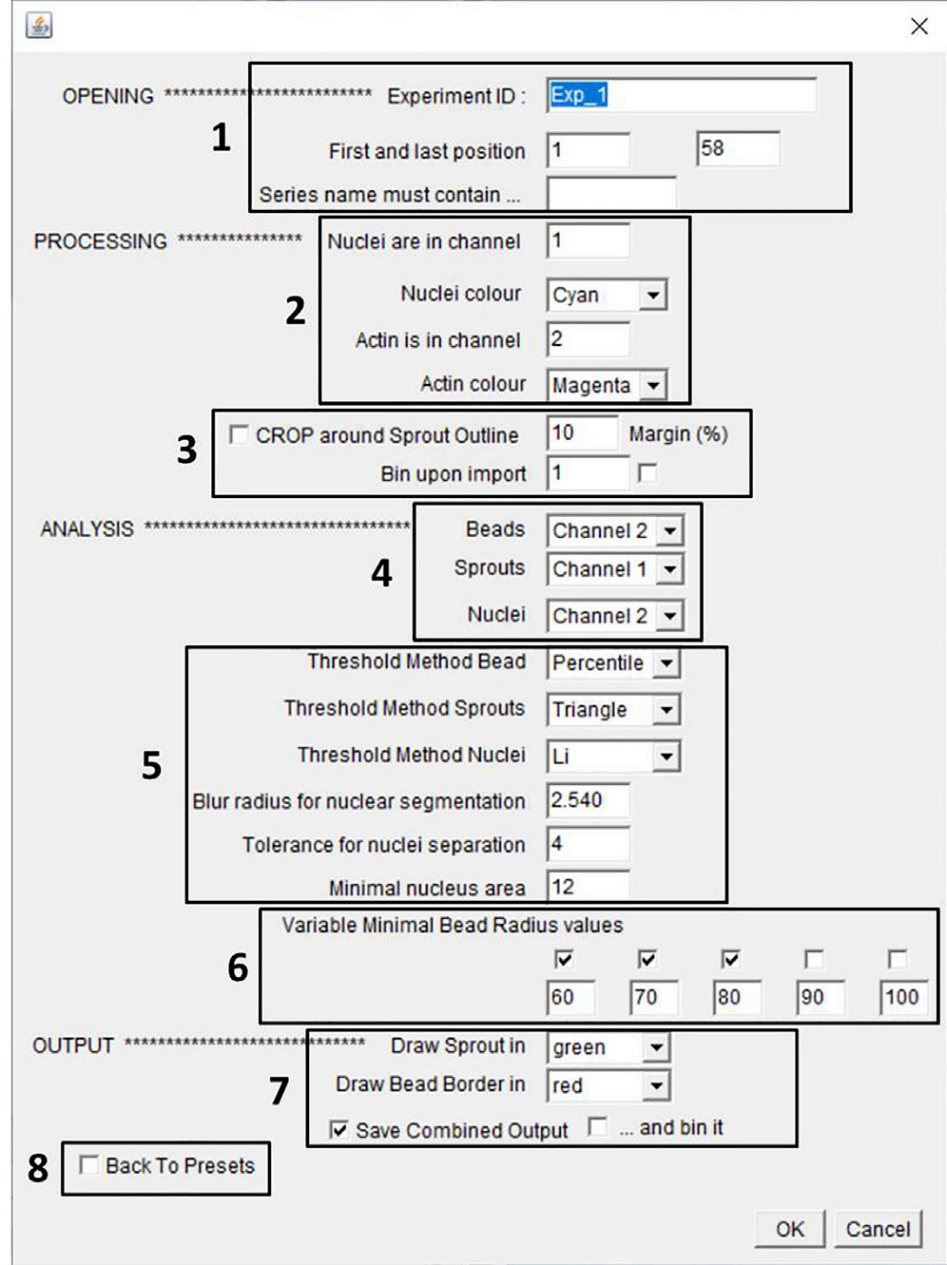
Screenshot of the settings window from our “*Automated sprout analysis*” macro Appropriate settings for the opening, processing, analysis and output of confocal z-stacks of sprouting can be defined here, as described in the main text.
  g. Name the experiment and select the positions to be analyzed (*1* in Figure 6)
  h. Define staining and colors of separate channels (*2*)
  i. If necessary, select options to reduce RAM size, e.g., cropping around the entire sprout outline and/or binning (*3*).
    ***Note:*** Binning will speed up analysis and use less memory, but be aware that reduced resolution may impact the results
  j. Define which channels to use for detection of nuclei, beads and sprouts (*4*)
  k. Select thresholding settings and options for optimal detection of individual nuclei (segmentation) (*5*). Standard settings used in this paper are shown.
    ***Note:*** Appropriate settings for sprout and bead thresholding should be determined and optimized first for individual experiments.
  l. Select the minimal bead radius (MBR), ranging from 60 to 100 μm (*6*).
    ***Note:*** Varying the MBR will affect the detection of the bead, and thereby also the numbers of nuclei in sprouts and sprout length. It is advised to first try all MBRs to determine the optimal value in order to get the best size and most circular shape, which will be clear from the created output file (Figures 7F–7K) (troubleshooting 8).
    **Figure 7:**
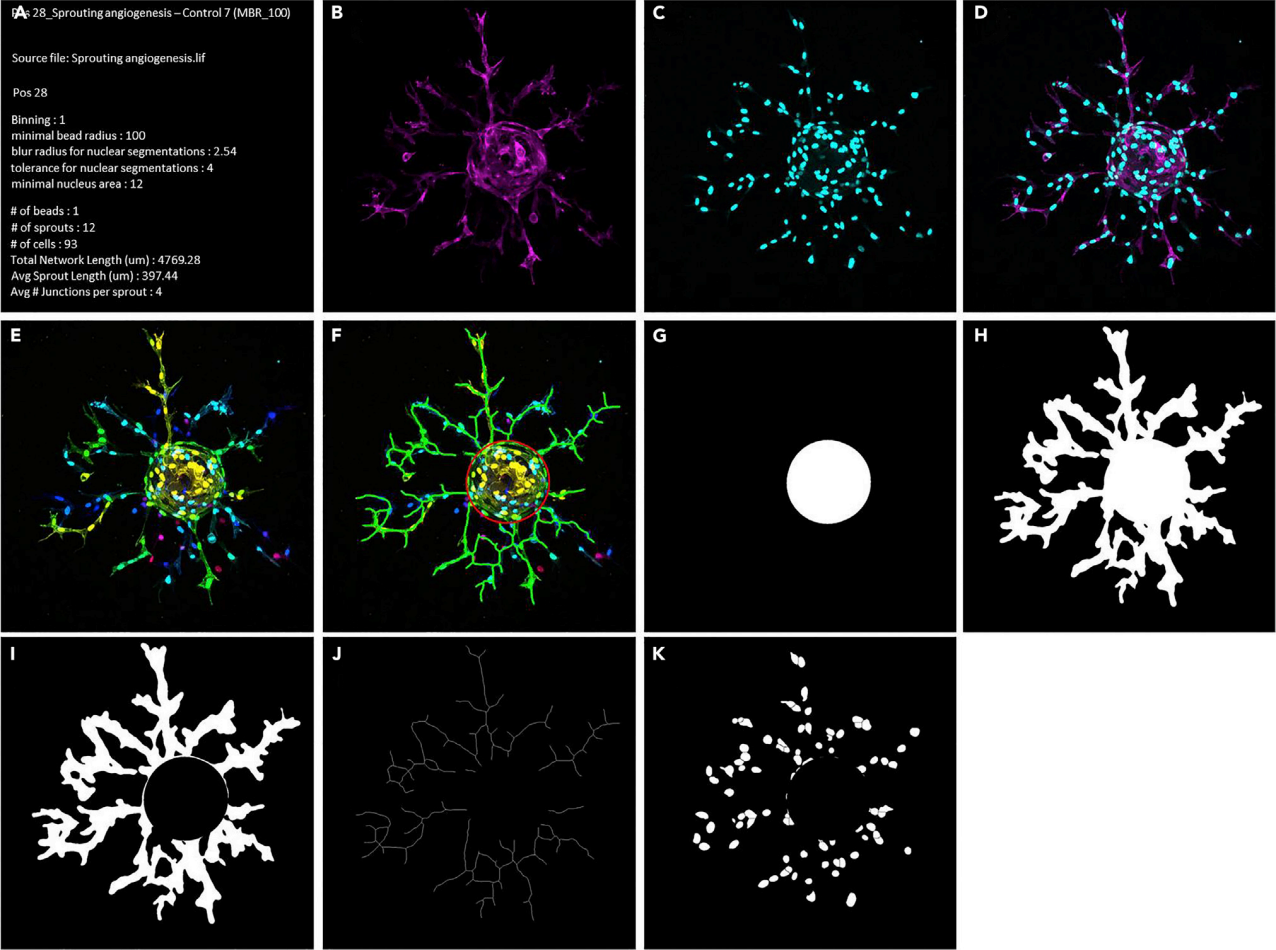
Example of an output file The macro described here will generate for each individual bead (position) a file containing the analysis settings and main results (A), the separate channels and merge of the maximum projection (B–D), a depth-coded image with and without the outlined bead and spouts (E and F), the bead and sprout masks (G–I), skeletonized sprouts (J), and the segmented nuclei (H). The image stack used here contained 362 z-slices, separate channels were F-actin (*magenta*) and nuclei (*cyan*), and depth coding was performed with the LUT *Spectrum*.
  m. Select settings for the output file, such as colors of the bead and sprout outlines (*7*). The “*save combine output*” option prompts the generation of a tiff combining all analyses from the current experiment in a multidimensional format, designed to facilitate evaluation of the results (see 3a below). To reduce output file size, use the “*bin it*” option (this option leaves data analysis itself unaffected).
  n. Finally, the option to return to the pre-defined settings can also be selected (*8*)
    ***Note:*** Output data are saved to a folder structure, organized by Experiment ID. On first use, the user will be prompted to select the destination for the output folder, which will from then automatically be used in subsequent analyses.
    ***Note:*** The time necessary to complete analysis depends on the computer used. For the analysis of large data sets, a fast computer with sufficient RAM (at least 16 GB) is crucial (troubleshooting 8).

1. Install ImageJ plugin *Sprout morphology.*
  a. Menu bar → Help → Update…→ Manage update sites → Check Angiogenesis.
  b. Click close and apply changes.
  c. Restart ImageJ/FIJI.

2. Run macro.
  a. Import “*Automated sprout analysis*” macro in ImageJ
  b. Click Run > the macro will open the window “*macro to reiterate sprouting analysis*” > click OK
  c. The macro will open a window to select the appropriate file > click OK
  d. The macro will open the “*Bio-Formats options*” window > select the appropriate settings (*Virtual Stack* recommended) > click OK
  e. Macro will then open the window “*Bio-Formats series options*” > select a single position (the sole purpose here is readout of metadata; user can later define positions to be analyzed) > click OK
    ***Note:*** Bio-Formats will allow direct import of raw data without the need to export them first from the microscopy software. Bio-Formats will retrieve the appropriate/relevant dimensions directly from the metadata.
  f. Macro will open a Dialog Window to define all desired settings (see Figure 6). Pre-defined settings are shown (and can be retrieved later).

3. Analyze data.
  a. The macro will store the generated results on the selected drive, in a folder called “*Results Sprouting Analysis*”. Data from each experiment will be stored in a separate folder, and will contain (among others) a tiff file with two sliders. The bottom slider will enable toggling between all individual positions (beads of the experiment, while the top slider will enable scrolling through all analysis results (see Figure 7), including the name of experiment/condition as well as used settings and obtained results (A), the separate and merged channels of the max projection (B-D), depth coding with and without outlined bead and sprouts (E,F), the bead and sprout masks (G-I), skeletonized sprouts (J), and the segmented nuclei (H).
  b. The macro will also save a basic Excel (xls) file, containing the used settings as well as the results from all positions (# of beads, sprouts, and nuclei, total network length, average sprout length and sprout width). For opening in Excel, choose semicolon as delimiter and use ‘.’ as Decimal separator and ‘’(empty) as Thousands separator.

***Note:*** Graphs can be created directly in Excel or using an alternative program such as GraphPad Prism. The latter also allows direct statistical analysis. Start from data table or graph → *Analyze* → choose the appropriate method of statistical analysis, depending on experimental set-up and number of conditions.

## Limitations

Technical problems may arise at several steps of the protocol described here, associated for instance with cell culture, sprouting efficiency, staining and multi-position imaging of fixed cells, live-cell microscopy, or the analysis of image stacks. Potential solutions to these issues are detailed in the troubleshooting section. It is important to realize that endothelial cells can differ from one individual to another, and that not all HUVECs proliferate and sprout to the same extent, which is a source of variability between different labs and/or different sets of experiments in the same lab. Moreover, this assay relies on generating confocal z-stacks from multiple different conditions in multi-position imaging mode, which requires an optimal balance between the number of z-slices per position, the number of positions to be imaged, and the resolution (and therefore speed) of imaging. Reductions in z-step size will lead to an increase in the number of z-slices and thus provide more detail, but will also require more imaging time. Also, higher resolution will decrease the speed of imaging and vice versa. Access to confocal microscopes and available imaging time may be limited, although for this type of experiments a positions list can be created at the end of the day to run the experiment for 12–16 h. During live imaging, limitations will include detrimental effects resulting from the use of fluorescent dyes and/or phototoxicity, as well as bleaching of the fluorescent signal over time. Furthermore, there are limitations to the number of z-slices and positions, as well as the time interval in between two consecutive positions, that can be imaged in a multi-position live experiment. Finally, the analysis of image stacks using ImageJ requires a fast device with sufficient memory, and can be painfully slow if a ‘regular’ computer is used.

## Troubleshooting

### Problem 1

HUVECs grow slower than expected (steps 20 and 21 of before you begin).

### Potential solution

Cell adhesion and proliferation can be enhanced by coating the flasks with gelatin, fibronectin, or serum. Furthermore, proliferation is optimal when cells are seeded at appropriate densities, i.e., not too sparse and not too dense.

### Problem 2

Viral titer is low (steps 32 and 33 of before you begin).

### Potential solution

To increase viral titer, try to enhance the efficiency of transfection. HEK293T cell adhesion and proliferation can be improved by coating the tissue culture dishes. HEK393T cells are best transfected at a density of 50%–80%. Low-passage HEK293T cells produce a higher titer than high-passage cells. Finally, the viral supernatant can be concentrated using PEG 6000 (Kutner et al., 2009). While viral supernatant can be stored at −80°C for a number of weeks to even months, best results are achieved using freshly isolated supernatant.

### Problem 3

Few cells are left after selection (step 7).

### Potential solution

The viral titer may be too low, which can be solved by optimizing the transfection procedure in HEK293T cells, or by concentrating the virus as described above. Alternatively, some shRNAs can cause cell death due to off-target effects. Comparison of different shRNAs per target gene is advised. Finally, if the gene of interest is an essential gene, its suppression may impair cell viability and/or proliferation. In this case it is likely that selection occurs for cells that have low levels of knockdown, and thus express residual levels of the protein that rescue compromised proliferation or cell viability.

### Problem 4

Gels do not polymerize well and/or are of poor quality (steps 15 and 16).

### Potential solution

The quality and relative amounts of the used fibrinogen and thrombin are essential. Thrombin can be aliquoted and maintained at −80°C, but its activity may decrease over time during prolonged storage. Furthermore, it is recommended to use high-purity fibrinogen preparations. Suboptimal quantities of active thrombin and/or fibrinogen will cause the gel either to polymerize too fast and/or become too rigid, which will impair sprouting, or not polymerize fast/good enough, in which case the beads may sink to the bottom of the well and the cells will start adhering to the tissue culture glass plate (see Figure 3).

### Problem 5

Excessive sprouting is observed after 48 h (hypersprouting), which can complicate analysis (see Figure 3) (step 17).

### Potential solution

Determine the optimal conditions in a pilot experiment. In case of excessive sprouting, reduce the time of the assay (fix the cells at an earlier time-point).

### Problem 6

Labeling with CellTracker induces toxicity, or dye transfer between adjacent cells is observed (steps 21 and 22).

### Potential solution

Optimal conditions for labeling and use of CellTracker dyes should be assessed first. Too high concentrations can reduce cell viability and performance, while too low concentrations, especially combined with bleaching effects during imaging, will compromise visibility. Moreover, transfer of dyes between adjacent cells can occur. Several dyes are available, fused to a variety of fluorophores and labeling distinct subcellular compartments, and comparison of these will identify the optimal dye for specific experimental conditions.

### Problem 7

The signal is bleaching, cells look unhealthy, or cells do not behave as expected in live imaging assays (step 35).

### Potential solution

The dyes may impair cell performance directly or as a result of phototoxicity during live imaging. First assess the direct effects of the dyes on cell function, by incubation during the same time-frame but without exposure to light. Then aim for the minimal concentration of dye that will still generate sufficient visibility even after long-term imaging. If impaired cell function results from phototoxicity, a solution may be to increase the interval between consecutive images (thus reducing the total number of images), as well as reduce the number of z-slices at each position. On a confocal microscope, resonant scanning may reduce bleaching effects and phototoxicity. Alternatively, other systems like light sheet microscopes could resolve these problems.

### Problem 8

The macro does not generate results or aborts analysis (steps 1 and 2 of quantification and statistical analysis).

### Potential solution

Allocating more RAM to ImageJ is recommended (*Edit* > *Options* > *Memory and threads*). If insufficient RAM is available, the macro will abort analysis. The user can resume analysis by selecting the appropriate position in the Settings Dialog. The macro also indicates when analysis for a given position failed. This is usually because the bead was not properly detected, and can be solved by selecting a different MBR.

## Resource availability

### Lead contact

Further information and requests for resources and reagents should be directed to and will be fulfilled by the lead contact, Coert Margadant (c.margadant@amsterdamumc.nl).

### Materials availability

This study did not generate new unique reagents.

### Data and code availability

The ImageJ macro “*Automated sprout analysis*” is available from GitHub (https://github.com/Sprouting-angiogenesis/Automated-sprout-analysis).
